# Non-inhibitory levels of oxygen during cultivation increase freeze-drying stress tolerance in *Limosilactobacillus reuteri* DSM 17938

**DOI:** 10.3389/fmicb.2023.1152389

**Published:** 2023-04-14

**Authors:** Nikhil Seshagiri Rao, Ludwig Ermann Lundberg, Julia Tomasson, Cecilia Tullberg, Daniel P. Brink, Shuai Bai Palmkron, Ed W. J. van Niel, Sebastian Håkansson, Magnus Carlquist

**Affiliations:** ^1^Division of Applied Microbiology, Department of Chemistry, Lund University, Lund, Sweden; ^2^The Department of Molecular Sciences, Uppsala BioCenter, Swedish University of Agricultural Sciences, Uppsala, Sweden; ^3^BioGaia, SE-103 64, Stockholm, Sweden; ^4^BioGaia, SE-241 38, Eslöv, Sweden; ^5^Division of Biotechnology, Department of Chemistry, Lund University, Lund, Sweden; ^6^Department of Food Technology, Engineering and Nutrition, Department of Chemistry, Lund University, Lund, Sweden

**Keywords:** online flow cytometry, k mean clustering, fatty acid, bile and acid tolerance, 5′ nucleotidase, *Limosilactobacillus reuteri* DSM 17938, oxygen, freeze-drying

## Abstract

The physiological effects of oxygen on *Limosilactobacillus reuteri* DSM 17938 during cultivation and the ensuing properties of the freeze-dried probiotic product was investigated. On-line flow cytometry and *k*-means clustering gating was used to follow growth and viability in real time during cultivation. The bacterium tolerated aeration at 500 mL/min, with a growth rate of 0.74 ± 0.13 h^−1^ which demonstrated that low levels of oxygen did not influence the growth kinetics of the bacterium. Modulation of the redox metabolism was, however, seen already at non-inhibitory oxygen levels by 1.5-fold higher production of acetate and 1.5-fold lower ethanol production. A significantly higher survival rate in the freeze-dried product was observed for cells cultivated in presence of oxygen compared to absence of oxygen (61.8% ± 2.4% vs. 11.5% ± 4.3%), coinciding with a higher degree of unsaturated fatty acids (UFA:SFA ratio of 10 for air sparged vs. 3.59 for N_2_ sparged conditions.). Oxygen also resulted in improved bile tolerance and boosted 5′nucleotidase activity (370 U/L vs. 240 U/L in N_2_ sparged conditions) but lower tolerance to acidic conditions compared bacteria grown under complete anaerobic conditions which survived up to 90 min of exposure at pH 2. Overall, our results indicate the controlled supply of oxygen during production may be used as means for probiotic activity optimization of *L. reuteri* DSM 17938.

## Introduction

Probiotics are defined as live microorganisms that when administered in adequate amount confer a health benefit on the host (Hill et al., 2014). The main probiotic bacteria having benefits to human health such as are from the group of lactic acid bacteria (LAB). Among these is *Limosilactobacillus reuteri* DSM 17938(previously named *Lactobacillus reuteri* 17,938; Zheng et al., 2020), which has been one of the industrially most relevant bacteria for well over 20 years. *Limosilactobacillus reuteri* DSM 17938 has demonstrated to attenuate human conditions such as abdominal pain, diarrhea (acute, infectious and nosocomial), infant colic, and necrotizing enterocolitis (Francavilla et al., 2012; Wanke and Szajewska, 2012; Roos et al., 2013; Oncel et al., 2014; Chau et al., 2015; Dinleyici et al., 2015; Weizman et al., 2016).

Commercial probiotics often consist of freeze-dried bacteria together with a lyo-protectant (e.g., sucrose). To confer the health benefit, the bacteria must resist any unfavorable conditions faced during production, as well as during storage, handling, and administration to the host. The physiological function of the bacteria depend on the environmental conditions, and primarily the fermentation step determine their robustness to freeze-drying (FD) stress (Palmfeldt and Hahn-Hagerdal, 2000; Schoug et al., 2008; Li et al., 2009; Liu et al., 2014; Velly et al., 2015) and subsequent adverse conditions (Hernández et al., 2019). In addition to FD stress tolerance, the industrial production ideally also considers additional probiotic activities. For example, the bacterium must also survive in the GI-tract, where they encounter gastric juice in the stomach and bile and pancreatic juice in the intestines (de Melo Pereira et al., 2018; Castro-López et al., 2022). Further, the pain-reducing properties of DSM 17938 is believed to be partly due to the antagonistic activity of the bacterium to the membrane bound nociceptor TRPV1 (transient receptor potential vanilloid 1; Perez-Burgos et al., 2015). Therefore, optimization of the production ideally takes into account TRPV1 modulating activity, which is believed to be associated with bacterial production of the enzyme 5′ nucleotidase (5′ NT) hydrolyzing AMP to adenosine (Puntambekar et al., 2004; Pang et al., 2022).

Oxygen is an important cultivation process control variable with potentially both negative and positive influences on the bacterium during cultivation and product formulation. On the one hand, *L. reuteri* lacks a functional proton-translocating respiratory chain and is sensitive to oxygen, so anaerobic conditions are typically applied during production (Papadimitriou et al., 2016). Low levels of oxygen may, however, be beneficial as supporting role in the keeping of redox homeostasis. In *L. reuteri,* NADH formed in the Embden-Meyerhof pathway and in the phosphoketolase pathway is re-oxidized by alcohol dehydrogenase and lactic acid dehydrogenase. Still, an excess of NAD(P)H is produced with the use of glucose as sole carbon and energy source, and the addition of an external electron acceptor is required to achieve efficient growth. Co-substrates, such as citrate and fructose, are typically used as supplements since they are readily reduced by NAD(P)H-dependent dehydrogenases (Årsköld et al., 2008; Strömberg et al., 2017). Oxygen may be a better redox sink through direct enzymatic oxidation of NADH, similarly as shown for other lactobacilli (De Angelis and Gobbetti, 2004). Pyruvate can then be converted to acetate yielding an extra ATP per glucose instead of recycling NADH through formation of ethanol and lactic acid (Condon, 1987; Gänzle, 2015).

Oxygen has also been shown to be involved in the formation of unsaturated fatty acids in the phospholipid membrane in several bacterial species (Zhang and Rock, 2008; Nachtschatt et al., 2020). A high degree of unsaturated bonds results in higher membrane fluidity (Liu et al., 2014), which has been shown to have a positive effect on FD tolerance (Fonseca et al., 2019). Simple cultivation process control methods to increase membrane fluidity are therefore highly desirable. In contrast to other *Lactobacillaceae* (Guerzoni et al., 2001; Lin, 2006), *L. reuteri* does not have any known fatty acid desaturases, and unsaturated bonds are formed through the fatty acid synthase complex system, partly described for anaerobic bacteria before (Keweloh and Heipieper, 1996; Subramanian et al., 2010; Zhang and Rock, 2016). Whether oxygen influences membrane composition in desaturase-negative lactobacilli is still unclear.

Here, the effect of different levels of oxygen on cell growth, central carbon metabolism, lipid membrane composition, and FD tolerance was investigated for *L. reuteri* DSM 17938. The strain DSM 17938 was chosen as representative strain for the species with significant interest due to its probiotic activities. Online flow cytometry (FCM) using a previously developed OnCyt module (Besmer et al., 2014), and a *k*-means clustering-based gating method, were performed to get a detailed picture of effects on cell proliferation and viability. In parallel, the effect of low oxygen on acid tolerance, bile tolerance, and 5′ NT activity, which are important characteristics and markers for probiotic activity (Ruiz et al., 2013; Wang et al., 2018; Pang et al., 2022; Wendel, 2022), were also investigated.

## Materials and methods

### Microorganism and growth medium

*Limosilactobacillus reuteri* DSM 17938 provided by BioGaia AB, was used for all the experiments. Working stocks were stored in 15% (v/v) glycerol at −80°C. De Man-Rogosa-Sharpe (MRS) media (Merck, Germany) were used for all the cultivations throughout the work.

### Bioreactor cultivations

Pre-cultivation was conducted in two steps. A cryo-vial from the working stock was thawed in a water bath at room temperature for 3 min. The inoculum was prepared in 15 mL Falcon tubes containing 10 mL MRS media inoculated with 100 μL of the working stock solution and were incubated for 6 h at 37°C. Subsequently, 500 μL from the 10 mL preculture was inoculated to 50 mL Falcon tube with MRS media and incubated at 37°C for approximately 16–20 h. This was used as the inoculum for the bioreactor with a start OD620 of 0.2.

The bioreactor cultivations were performed at 1 L scale in 3 L Minifors-2 bioreactor system (Infors HT, Switzerland) equipped with eve® software (Infors HT, Switzerland, version 1.92.498.1). The cultivations were performed at 37°C, maintaining a stable pH at 6.00 by using 3 M potassium hydroxide, continuous stirring speed of 250 RPM and with three different levels of controlled aeration (500 mL/min of air sparging, no sparging and 500 mL/min of N_2_ sparging). Cultivations were performed in triplicates for each aeration condition. Samples for optical density, high performance liquid chromatography (HPLC) were drawn every hour in addition to the samples drawn directly from the online flow cytometer. The optical density was measured at 620 nm (OD_620_) using a U-1100 spectrophotometer (Hitachi, Japan). Cell dry weight was measured at harvest. To determine the cell dry weight (CDW), a sample of 10 ml culture volume was taken and filtered through a pre-weighed Supor-200, 0.2 mm filter (PALL Life Sciences, Mexico) in duplicate. The filters were then washed three times and left to dry in a desiccator overnight. The dry filters were subsequently weighed on an analytical balance (Mettler Toledo, Ohio, United States).

### Online flow cytometry

Online flow cytometry was based on OnCyt (OnCyt, Switzerland), coupled to a BD Accuri C6+ flow cytometer (BD Biosciences, United States), as described by Besmer et al. (2014). Samples were automatically drawn directly from the Minifors-2 bioreactor system (Infors HT, Switzerland) with a sampling frequency of *ca.* two samples per hour and culture. Samples were diluted in 10 mM TRIS buffer, pH 8 (Merck, Germany), and stained with a mix of SYBR Green I (Invitrogen, United States) and propidium iodide (PI; Sigma-Aldrich, Switzerland), incubated at 37°C for 10 min and analyzed in BD Accuri C6 plus flow cytometer. The laser utilized had an excitation wavelength of 488 nm. The fluorescence emission levels were measured with a band pass filter at 530/30 nm (FL1, SYBR® Green I) and a long pass filter at >670 nm (FL3, PI). The sample volume collected was 20 μL, with a medium flow rate of 35 μL/min. To record fluorescence for SYBR® Green I and PI, a threshold of 500 channel number (chnr) was applied on FL1-H (533 ± 30 nm), while no threshold was used on FL3-H (> 670 nm). The OnCyt dilution regime was set to 1,000-fold during the cultivation. A cleaning cycle with hypochlorite solution (0.5% active chlorine), quenching solution (10 mM sodium thiosulphate) and 2 runs of milliQ water were used between runs to avoid contamination from the previous sample. To ensure that a fresh sample was collected, 2 ml was drawn from the bioreactor and sent to the waste before each sampling.

### Flow cytometry data processing: Gating strategy

Automated gating to enumerate total cells and subpopulations observed in the bivariate plot of FL1-H and FL3-H (total cell count, viable cells, damaged cells A, damaged cells B, and cell debris were identified based on different fluorescence intensity of SYBR Green and PI) was developed for MATLAB R2019a. A *k*-means clustering was made with the MATLAB function *kmeans*, with the distance type set to “cosine,” and the number of replicates for each sample set to 20. The *k*-means clustering method was compared with (i) fixed gating based on user-defined linear equations in MATLAB, as well as (ii) manual “sample-by-sample” gating using the FlowJo v10.7 software. The gating strategies are elaborately described and compared in Supplementary material S1. The two gating methods performed in MATLAB correlated well with the manual gating method using FlowJo (Correlation coefficient R^2^ > 0.9–0.99). The *k*-means method was deemed more suitable for unbiased data processing since it needed the least manual input by the user, and still captured the dynamics of the FCM data. Therefore, the results presented in the paper are based on *k*-means method. The MATLAB code used to process and analyze the flow cytometry data is available on the following GitHub page: https://github.com/MicrobialEngineeringGroupTMB/.

### Freeze drying procedure

Cells were harvested when the base (3 M KOH) consumption stopped. At the end of each cultivation, the cells were harvested by centrifugation (Eppendorf 5810R, Germany) at 3,000 × g for 10 min. The supernatant was discarded, and the cell pellet was resuspended in 20% sucrose solution (final concentration—10%). Prior to freezing, resuspended cells were aliquoted in 8 mL Schott FD vials (filled to 2 mL), and the vials were covered with a rubber cap. FD was performed with Epsilon 2-6D LSCplus (Martin Christ, Germany). FD cycle was performed similar to Rao et al. (2021). In brief, the pressure was decreased to 0.072 mbar and shelf temperature increased to −38°C with a rate of 0.13°C/min during primary drying. Secondary drying was conducted at the same pressure as the primary drying and the shelf temperature was increased to 20°C with a rate of 0.083°C/min.

### FD tolerance

Freeze dried bacteria were rehydrated by resuspended in equal amount of phosphate buffered saline (PBS; 2 mL/vial) and incubated at room temperature for 20 min. The number of viable cells were determined before and after freeze-drying by plating (CFU counts) on MRS agar (Merck) which were incubated for 48 h anaerobically at 37°C. FD survivability is defined as ratio between viable cells after and before FD stress (expressed in %).

### Low pH tolerance assay

Freeze dried bacteria were resuspended in equal amount of PBS (2 ml/vial) and incubated at room temperature for 20 min. Ten μL was suspended in 10 mL of synthetic gastric juice (8.3 g/L proteose peptone, 3.5 g/L glucose, 2.05 g/L NaCl, 0.6 g/L KH_2_PO_4_, 0.11 g/L CaCl_2_, 0.37 g/L KCl, adjusted to pH 2.0 with HCl; Wall et al., 2007) and allowed to incubate (standing still) in a water bath at 37°C. Samples were taken at timepoints 0, 20, 50, and 90 min after which serial dilutions and plating on MRS agar (Merck) were performed. The agar plates were incubated for 48 h anaerobically at 37°C after which colonies were counted.

### Bile tolerance assay

Freeze dried bacteria were resuspended in equal amount of PBS (2 mL/vial) and incubated at room temperature for 20 min. After diluting the samples with PBS to 10^8^ CFU/ml, 30 μL was suspended in 270 μL of MRS medium (Sigma Aldrich, Germany) + 0.5% bovine bile (Sigma, B3883; Hernández et al., 2019) in a BioScreen microtiter plate followed by incubation at 37°C in BioScreen © (Oy. Growth Curves AB, Helsinki, Finland). OD measurements were taken every 15 min and prior to measurement a shaking step of 30 s were inserted. The analysis was run for 24 h.

### 5′ nucleotidase activity assay

5′ nucleotidase (NT) converts adenosine monophosphate (AMP) to adenosine that can act as an effector molecule (Puntambekar et al., 2004; Pang et al., 2022). The 5′ NT activity of supernatants from the cultivations were measured using a 5′NT assay kit (Crystal Chem High Performance Assays, United States) according to the manufacturer’s instructions. The samples were performed in technical triplicates for 2 biological replicates.

### HPLC analysis

Samples were withdrawn at start and throughout the entire cultivation (once per hour). The liquid fraction of the samples was separated using 0.22 μm filter to get remove the cells. Concentrations of glucose, acetate, lactate, ethanol, citrate, and succinate were determined using HPLC (Waters, Milford, MA, United States) equipped with an Animex HPX-87H ion exchange column (7.8 mm × 300 mm, Bio-Rad, Hercules, United States) at 60°C using a mobile phase of 5 mM H_2_SO_4_ with a flow rate of 0.6 mL/min. Product yields were obtained from the net amount of product formed divided by the overall amount of sugar consumed. The specific consumption/production rate was obtained from the net amount of product formed/substrate consumed divided by the cell dry weight and time. Carbon balance (%) was calculated (end-point) as described previously (de Vrije et al., 2007).

### Lipid extraction and analysis of free fatty acid

Lipids were extracted, methylated and analyzed from the freeze-dried material containing *L. reuteri* based on Bligh and Dyer (1959) and modified according to Lindberg Yilmaz et al. (2021). In brief, freeze-dried bacteria were initially rehydrated up to 2 mL milliQ water to which 1.25 mL 0.15 m acetic acid, 3.75 mL methanol:chloroform (2:1 v/v) and 1.25 mL chloroform was added. The chloroform phases were recovered, dried under N_2_ gas. Samples were methylated by addition of methanol:sulfuric acid mix and incubate at 90°C for 30 min. MilliQ water was added to stop the reaction. Methylated FA were analyzed using Trace 1300 GC-FID with autosampler (Thermo Scientific). Data were analyzed using the Chromeleon Chromatography Data System (CDS) software (v7.2.10). The fatty acid methyl esters (FAME) identified were confirmed and quantified using external standard mix GLC 463 (Nu-Chek Prep, Inc., Elysian, United States) and heptadecanoic acid (C17:0) as internal standard. The FAME was expressed as μg of FAME per mg of cell dry weight (CDW). The relative abundance (%) was calculated by dividing the individual FAME by the total. The standard deviation on the total FAME was calculated by taking a square root of the sum of the variances. The UFA:SFA ratio was calculated as previously described (Schoug et al., 2008; Liu et al., 2014), considering the mean values of UFA and SFA. Statistical analysis to compare the significant differences between the total yields between the conditions and the ratio of UFA to SFA was performed using one-way ANOVA (Tukey’s multiple comparison test) in GraphPad Prism (v9.2.0) assuming normal distribution (passed Shapiro–Wilk test).

### Bioinformatic analysis of fatty acid desaturases

The currently available *L. reuteri* protein sequence datasets were analyzed for the presence of fatty acid desaturases using three different approaches. Firstly, the amino acid sequence of the fatty acid desaturase desA (Uniprot: O34653) from the model Firmicutes species *Bacillus subtilis* was used to search for potential homologous protein sequences in the *L. reuteri* data of the NCBI database^1^; desA has been reported to be essential for formation of unsaturated fatty acids (Aguilar et al., 1998). The analysis was run with blastp (Camacho et al., 2009) with the “Organism parameter set to *Lactobacillales (taxid:186826)*, *Lactobacillus (taxid:1578)*, or *Limosilactobacillus reuteri (taxid:1598)*, and the Database parameter set to *Non-redundant protein sequences (nr)*. No significant hits were found. Secondly, an inventory of all proteins currently annotated as fatty acid desaturases in the Uniprot database (The Uniprot Consortium, 2019) was made for the following taxonomic range: Firmicutes, Bacilli, Lactobacillales, *Limosilactobacillacae*, *Limosilactobacillus*. Lastly, the resulting amino acid sequences for all annotated genes in the *L.* reuteri SD2112 genome—the parental strain of DSM 17938—were subjected to a protein domain analysis using IntroProScan [v5.52-86.0; (Jones et al., 2014)]. All results of the bioinformatic analyses are available in Supplementary material S2.

### Statistical analysis

Experiments were performed in biological triplicates. One- and two-way ANOVA was applied on the data using software GraphPad Prism (v9.2.0) as specified in the relevant sections to derive statistical differences between the conditions.

## Results

### Oxygen influences growth, morphology, and metabolism during cultivation

**Figure 1:**
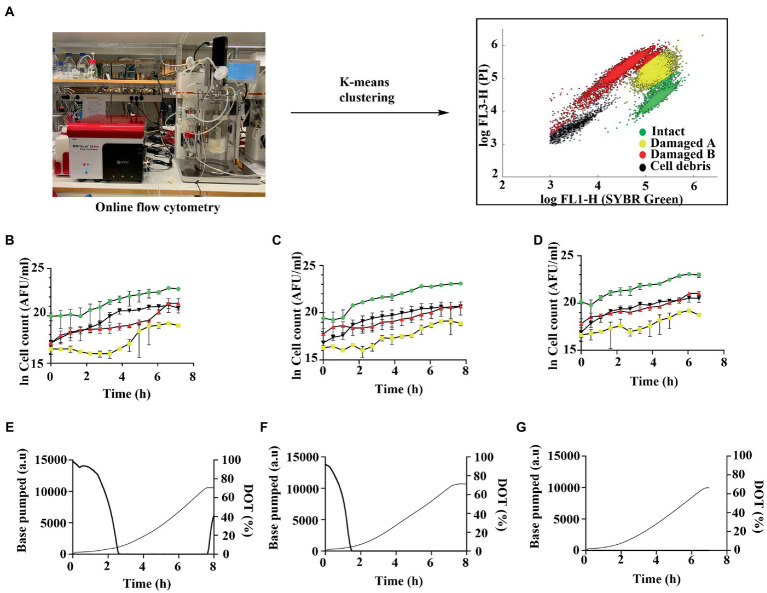
**(A)** Overview of experimental setup for online flow cytometry analysis of batch cultivation of *Limosilactobacillus reuteri* DSM 17938 in bench scale bioreactors using the OnCyt module connected to a BD Accuri C+ flow cytometer. Cell samples were diluted and stained automatically with SYBR green (FL1-H—533 nm) and propidium iodide (FL3-H—670 nm), enabling the distinction of four subpopulations: live cells (green), damaged cells A (yellow), damaged cells B (red), and cell debris (black). A *k*-means clustering method was developed for automated data processing and gating of subpopulations. Cell counts of subpopulations during **(B)** air-sparged, **(C)** non-sparged and **(D)** N_2_-sparged cultivations. Representative graph displaying dissolved oxygen tension (DOT%; bold line) and base consumption (thin line) **(E)** air-sparged, **(F)** non-sparged and **(G)** N_2_-sparged cultivations. Data are mean ± SD of three biological replicates for three conditions. AFU, active fluorescent unit; FL, fluorescence.

To study the effect of oxygen on *Limosilactobacillus reuteri*, DSM 17938 cultivations with three different levels of aeration were made in bench-scale bioreactors: (i) air-sparged; (ii) non-sparged; and (iii) nitrogen gas (N_2_) sparged. It was previously found that the bacterium tolerates aeration of up to at least 500 mL/min without negative impact on growth (Larsson et al., unpublished), which was the criterium for setting the higher oxygen level. The non-sparged condition was set as an intermediate level of oxygen supply compared to N2-sparged anaerobic condition. Growth was monitored by online FCM as well as conventional cultivation output parameters (e.g., base addition, OD, and CDW). Four different subpopulations were observed in the flow cytometry analysis: (i) intact cells, (ii) damaged cells A, (iii) damaged cells B, and (iv) cell debris (Supplementary Figure S1). To simplify the analytical pipeline and take advantage of the full potential of the extensive amount of data obtained by online FCM we developed a protocol for automated gating and generation of key output variables (total cell count, intact cells, damaged cells, and debris; Figure 1; c.f. Supplementary material for details). The gating protocol was based on *k*-means clustering, similarly to previously described for FCM analysis of other microorganisms (Aghaeepour et al., 2011; Ge and Sealfon, 2012), thereby minimizing the user bias associated with manual gating (Lencastre Fernandes et al., 2011).

**Figure 2:**
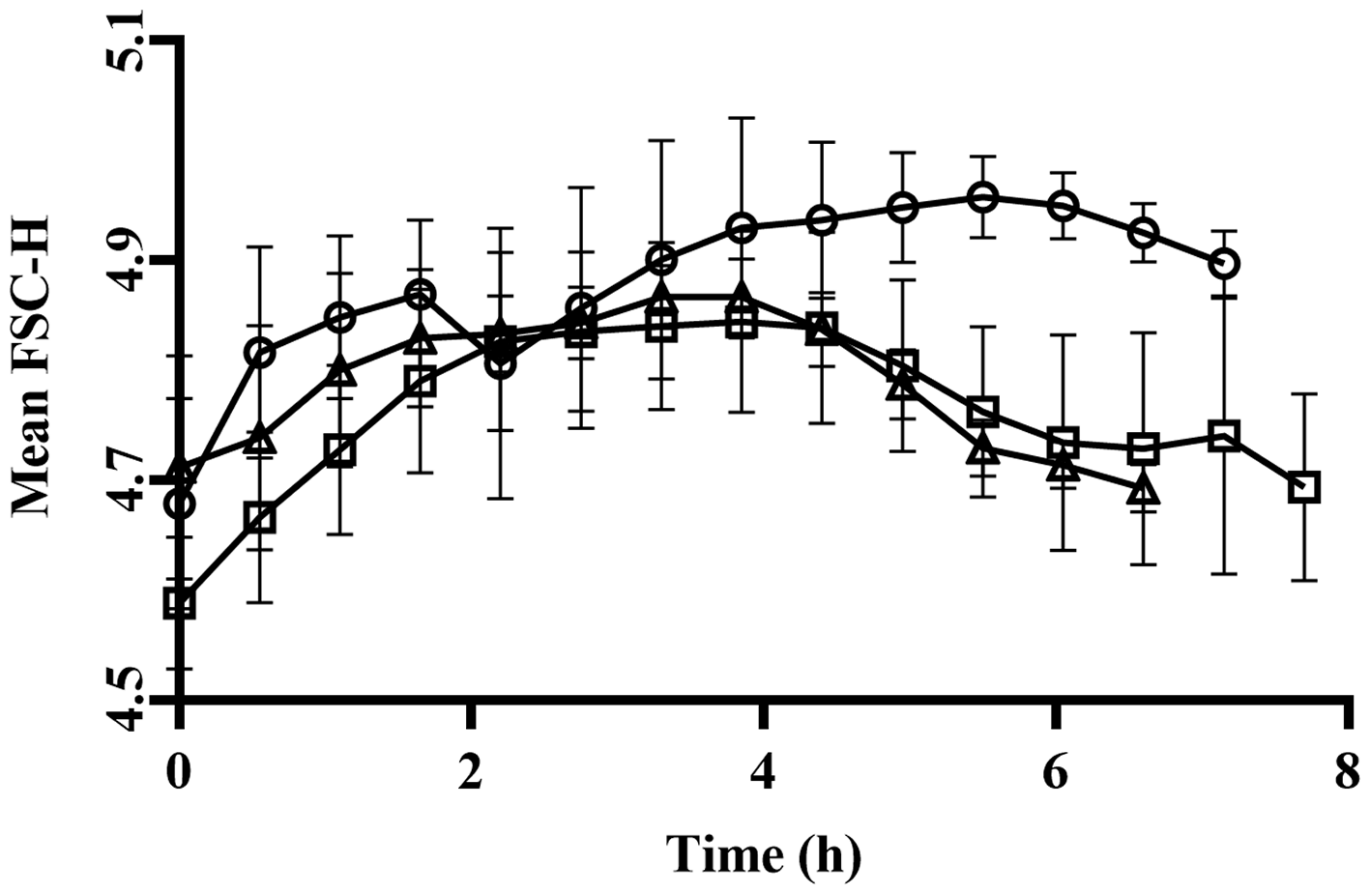
Mean forward scatter (FSC-H) profiles of *Limosilactobacillus reuteri* DSM 17938 during cultivation in air sparged, non-sparged or N_2_ sparged cultivations. Air sparged (open circle), non-sparged (open square) and N_2_ sparged (open triangle) Data are mean ± SD of three biological replicates for three conditions.

The highest specific growth rate (*μmax*) was observed for the cultivation sparged with N_2_ (0.799 ± 0.093 h^−1^) and lowest for the cultivation sparged with air (0.742 ± 0.129 h^−1^), although the difference was not statistically significant [*p* > 0.05; two-way ANOVA (Tukey’s multiple comparisons test); Table 1]. At harvest, which occurred 7–8 h after inoculation for all the cultures, the cell dry weight (CDW) were similar for the different conditions demonstrating that the applied oxygen levels did not significantly impact growth [air sparged:1.56 ± 0.11 g/L; non-sparged:1.62 ± 0.11 g/L; N_2_ sparged: 1.53 ± 0.03 g/L; *p* > 0.05; two-way ANOVA (Tukey’s multiple comparisons test)]. The trends in forward scatter signal obtained from FCM were similar for all the three sparged cultivations, with lower values at start and in the end of cultivation. Thus, there seem to be a small but detectible distinction of the cell morphology phenotype during the cultivations (Figure 2). Nevertheless, the final biomass amount both in terms of number of cells (AFU/mL) as well as OD_620_ was not statistically (*p* > 0.05) [two-way ANOVA (Tukey’s multiple comparisons test)]. Oxygen present in the media was measured using a dissolved oxygen tension (DOT) probe. In the non-sparging condition oxygen reached zero by 1.39 ± 0.10 h, whereas for air sparged condition 2.55 ± 0.06 h (Figures 1E–G). For the air sparged culture, after glucose depletion at time point between 7 and 8 h, the DOT start to increase until the measurement stopped due to harvest. This demonstrates that an active metabolism is needed to assimilate oxygen in the bulk liquid.

**Table 1:** Results of the batch cultivations with air sparging, no air and nitrogen sparging for *Limosilactobacillus reuteri* DSM 17938.

Condition	μ_max_ (h^−1^)		Yield (g.g glucose^−1^)				Specific productivity/consumption rate (g.gCDW^−1^ h^−1^)				Cell dry weight (CDW) (mg/mL)	Carbon balance (%)
	Calculated from OD	Calculated from FCM	Biomass	Acetate	Lactate	Ethanol	Acetate	Lactate	Ethanol	Glucose		
Air sparged	0.64 ± 0.03^a^	0.74 ± 0.13^a^	0.09 ± 0.00^a^	0.11 ± 0.01^a^	0.50 ± 0.01^a^	0.24 ± 0.01^a^	0.15 ± 0.01	0.68 ± 0.01	0.32 ± 0.02	1.36 ± 0.03	1.56 ± 0.11^a^	93.7 ± 0.3
Non-sparged	0.75 ± 0.02^a^	0.77 ± 0.14^a^	0.10 ± 0.00^a^	0.08 ± 0.00^a,b^	0.50 ± 0.01^a^	0.30 ± 0.03^b^	0.10 ± 0.00	0.64 ± 0.01	0.39 ± 0.03	1.28 ± 0.01	1.62 ± 0.11^a^	97.7 ± 2.6
N_2_ sparged	0.77 ± 0.03^a^	0.80 ± 0.09^a^	0.10 ± 0.00^a^	0.07 ± 0.01^b^	0.48 ± 0.01^a^	0.32 ± 0.00^b^	0.10 ± 0.01	0.70 ± 0.01	0.47 ± 0.01	1.49 ± 0.03	1.53 ± 0.03^a^	97.4 ± 1.7

Data are mean ± SD of three biological replicates for each of the three sparging conditions. Two-way ANOVA (Tukey’s multiple comparison test) to determine statistical significance. Equal letters indicate no statistical difference between the samples (*p*-value > 0.05). OD, optical density; FCM, flow cytometry.

**Figure 3:**
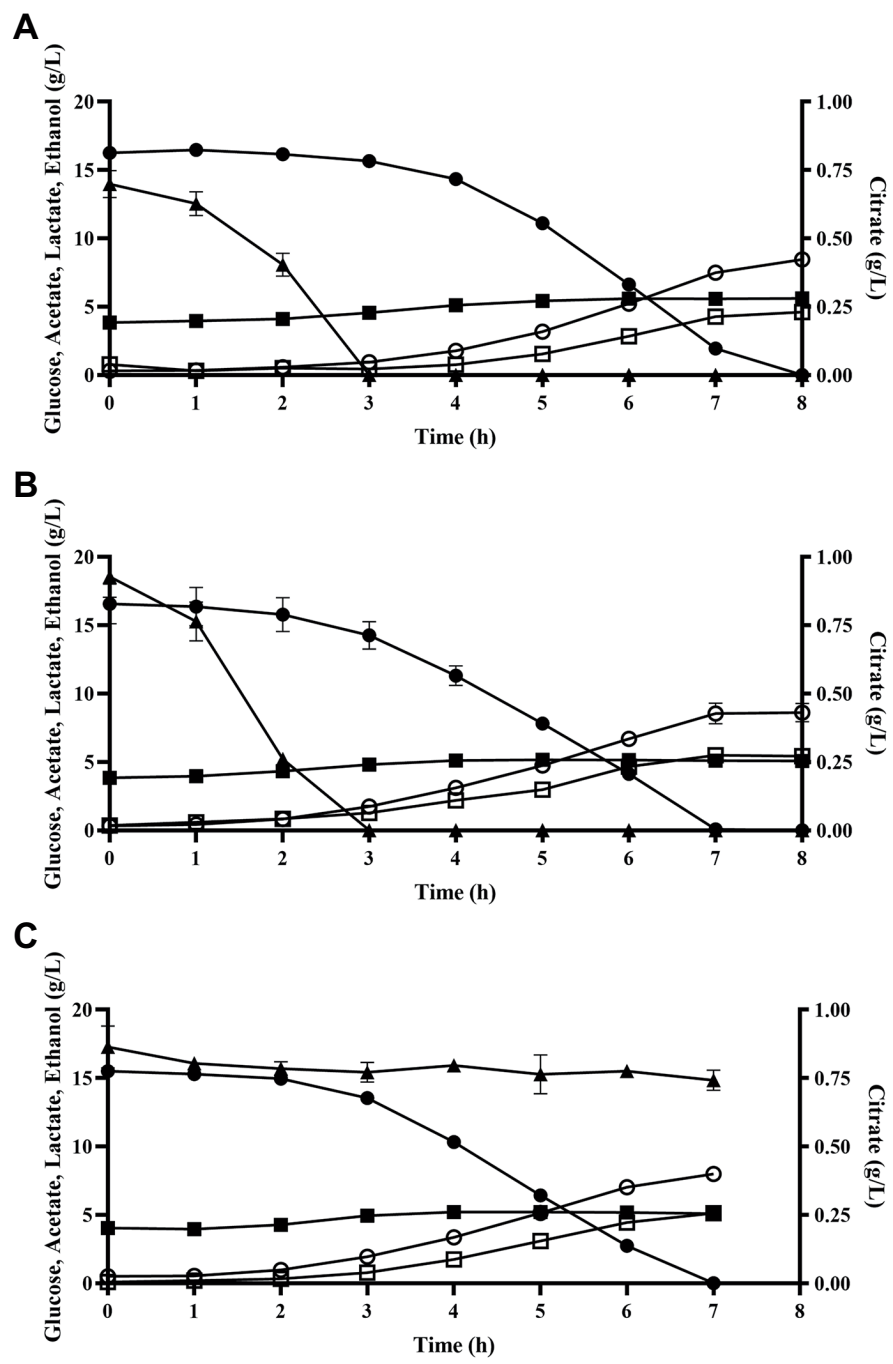
Metabolic profile for batch cultivation in 2 L bioreactors for the three conditions. **(A)** Air sparged, **(B)** non-sparged, and **(C)** N_2_ sparged. Data are mean ± SD of three biological replicates for 3 conditions. Glucose (filled circle), acetate (filled square), lactate (open circle), ethanol (open square), citrate (filled triangle).

Oxygen had a noticeable effect on the cell metabolism as substantiated with differences in the metabolite profiles between the three different aeration conditions (Figure 3). Glucose consumption rate was similar between cultures, reflecting the similar growth rate, but the yield of acetate in the air sparged cultivation was found to be 1.5× higher than for the non-sparged and N_2_ sparged conditions (value of *p* >0.05 and value of *p* = 0.0483, respectively; Table 1). The non-sparged and N_2_ sparged conditions instead yielded higher amounts of ethanol compared to air sparged condition (value of *p* = 0.0006 and value of *p* <0.0001, respectively). There was no significant difference for lactate production between the three conditions [value of *p* >0.05; two-way ANOVA (Tukey’s multiple comparisons test)]. Another difference was observed for citrate, which was not catabolized in the N_2_ sparged condition, whereas it was completely depleted in the other two conditions at similar timepoint (3 h) during cultivation (Figure 3). There were no traces of succinic acid, indicating that citrate was catabolized further to lactate or acetate as has been reported for some other strains (Hugenholtz, 1993). Carbon balances closed at 93.7% ± 0.3%, 97.7% ± 2.6% and 97.4% ± 1.7% for the air sparged, non-sparged, and N_2_ sparged cultivations, respectively (Table 1).

### Aerobic condition resulted in higher FD survival

**Figure 4:**
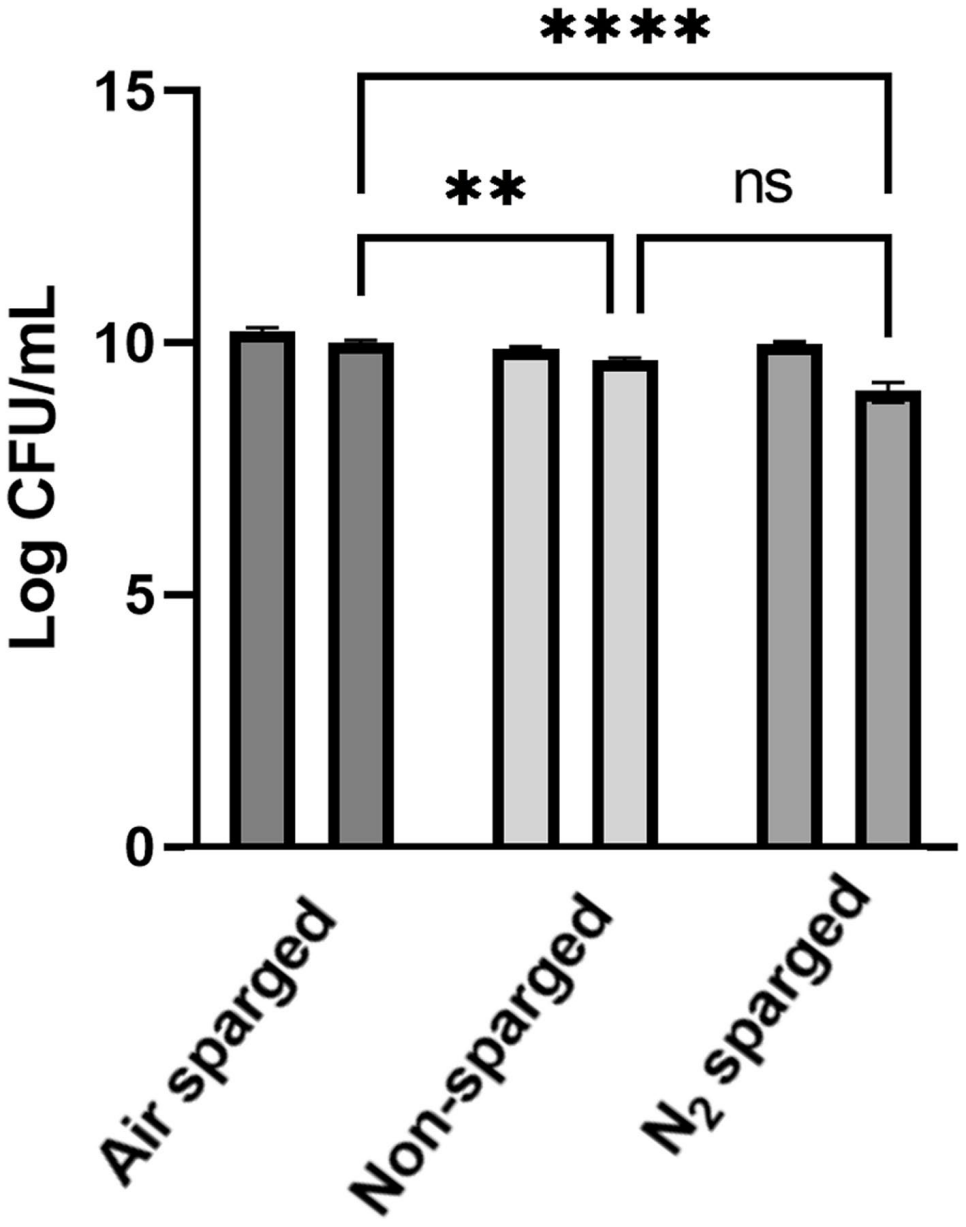
Reduction in cell count after freeze-drying FD stress. Two bars for each condition represents one for cell count before FD and other for cell count after FD. Statistical significance was calculated on FD survivability using two-way ANOVA (Tukey’s multiple comparisons test): ns, not significant, ** indicates value of *p* < 0.01, **** indicates value of *p* < 0.0001. Data are mean ± SD of three biological replicates for three conditions.

FD survivability is defined as ratio between intact cells after and before FD stress. The survival of *L. reuteri* DSM 17938 after FD was higher in the air sparged (61.8 ± 2.4%) and non-sparged conditions (60.5 ± 6.4%) compared to N_2_ sparged condition (11.5 ± 4.3%; Figure 4; Supplementary Table S2). In terms of reduction in cell count after freeze-drying FD stress, significant difference was observed between air sparged and non-sparged, N_2_ sparged conditions [value of *p* = 0.0048 and value of *p* <0.0001, respectively; two-way ANOVA (Tukey’s multiple comparisons test)]. In terms of FD survivability, there were significant differences between the air sparged, non-sparged condition and the N_2_ sparged condition (value of *p* = 0.0008 for both), and no significant difference was observed between the air sparged and non-sparged condition [*p*-value >0.05; two-way ANOVA (Tukey’s multiple comparisons test)].

### Oxygen improves bile tolerance and boosts 5′nucleotidase but displayed reduced tolerance to low pH

**Figure 5:**
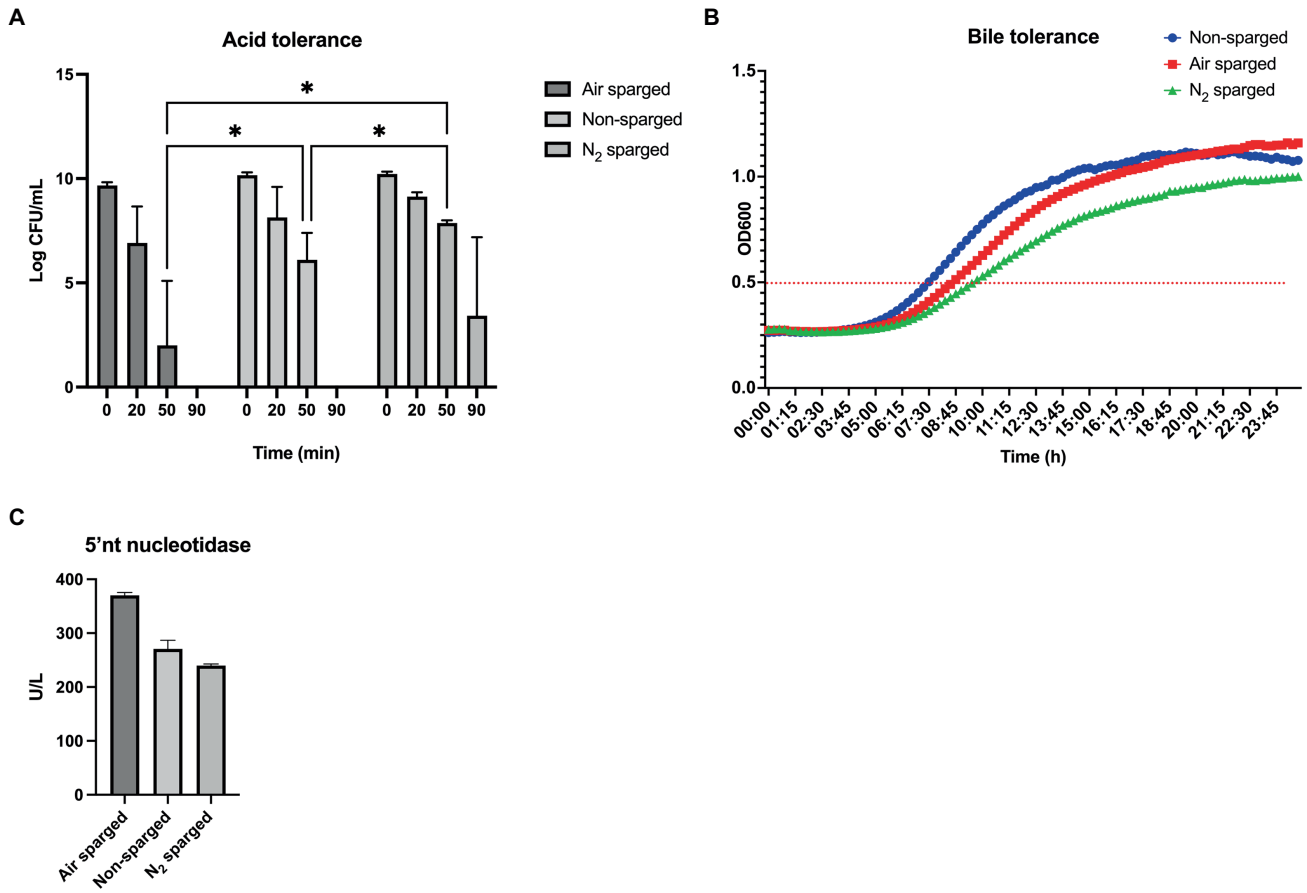
Probiotic functionality markers in FD products prepared for the three culture conditions. **(A)** Acid tolerance, **(B)** Bile tolerance and **(C)** 5′ nucleotidase (NT) activity. Statistical significance was calculated using two-way ANOVA for the bile tolerance and Friedman test with Dunn’s multiple comparisons test for the 5′NT activity. * indicates value of *p* < 0.05; ns, not significant. Data are mean ± SD of two biological replicates for three conditions.

The survival to low pH is a desirable probiotic trait since it is exposed to it in the stomach (gastric juice) and was evaluated for the three conditions. After exposure to pH 2.0 for 50 min, N_2_ sparged bacteria had survived significantly better than air sparged bacteria (value of *p* = 0.0128) and non-sparged bacteria (value of *p* = 0.0450; Figure 5A). In 3 out of 6 experiments, N_2_ sparged bacteria had survived at timepoint 90 min, while none of the other conditions allowed for survival at that timepoint, however this effect was not significant (*p* > 0.05).

Following exposure to low pH in the gastric pouch the bacteria will encounter bile in the intestine, making bile tolerance another important characteristic of probiotic bacteria. By cultivating the bacteria in MRS supplemented with 0.5% bovine bile and evaluating the time to reach OD 0.5 one can estimate how the different cultivation conditions affect the ability to tolerate and grow in presence of bile. The non-sparged condition resulted in the shortest lag time, taking 7 h 30 min before reaching OD 0.5, which was the cut-off optical density used to indicate the start of the exponential growth phase (Figure 5B). The air sparged bacteria took approximately 8 h 45 min to reach the cut-off, whereas the N_2_ sparged bacteria required 9 h 45 min (Figure 5B). Comparing this result with a control that was cultivated in MRS media alone resulted in lag time of 5 h for both non-sparged and N_2_ sparged conditions and 5 h 45 min for oxygen sparged condition (data not shown).

*Limosilactobacillus reuteri* DSM 17938 secretes the enzyme 5′-nucleotidase, which converts adenosine-monophosphate (AMP) into the potent signaling molecule adenosine (Strä and Strä, 2006). Sparging with air resulted in highest activity, reaching an average of 370 U/l, while no sparging had an activity of 270 U/L and the activity in N_2_ sparged bacterial supernatant was 240 U/L. The differences in activity were however not significant (Figure 5C).

### Oxygen during cultivation results in higher UFA:SFA ratio

High ratio of unsaturated to saturated fatty acid has been shown to have a positive effect on FD tolerance (Fonseca et al., 2019). Changes in oxygen levels induced modifications in the fatty acid composition of the cell membrane (Table 2). There was no significant difference between the total FA yield between the three conditions [*p*-value > 0.05; One-way ANOVA (Tukey’s multiple comparison test)]. Oleic acid was the predominant FA among all the identified FA in all the conditions (air sparged 64.39% ± 0.22%, non-sparged 50.13% ± 2.79%, N_2_ sparged 51.21% ± 1.70%). Palmitic acid was found at a relatively lower level in air sparged condition (7.94% ± 0.61%). The oxygen sparged condition resulted in a higher unsaturated fatty acid (UFA) to saturated fatty acid (SFA) ratio (10.00), which was significantly different compared to N_2_ sparged (3.59; value of *p* = 0.0009) and non-sparged condition (3.20; value of *p* = 0.0006). A protein domain analysis of genome of *L. reuteri* SD2112, the parental strain of DSM 17938, was used investigate the presence of fatty acid desaturases in *L. reuteri* (Supplemental material S2). No *B. subtilis* desA type desaturase domains were found (InterPro accessions: IPR005804, IPR012171). Two genes (NCBI accession numbers: WP_003671844.1, WP_003671852.1) were found to contain a fabA/fabZ domain (IPR013114, IPR010084), which has previously been related to formation of unsaturated fatty acids.

**Table 2:** Effect of different aeration conditions on the fatty acid composition of the cell membranes of *Limosilactobacillus reuteri* DSM 17938.

Identified and integrated FA	Air sparged		Non-sparged		N_2_ sparged	
	μg FAME/mg CDW	% Relative abundance	μg FAME/mg CDW	% Relative abundance	μg FAME/mg CDW	% Relative abundance
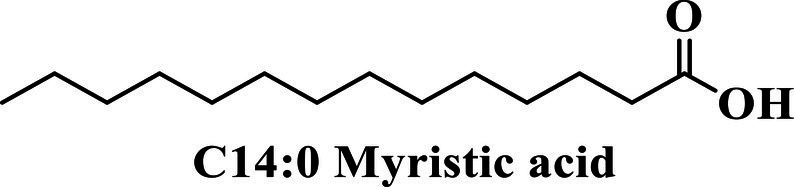	2.36 ± 2.07	1.01 ± 0.88	1.54 ± 1.37	0.73 ± 0.64	1.34 ± 1.20	0.69 ± 0.60
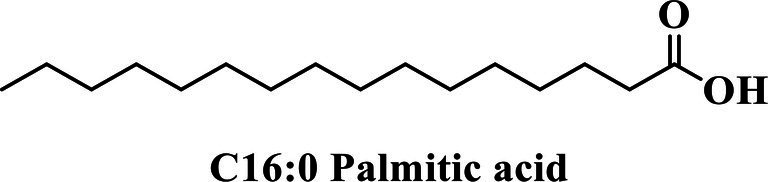	16.99 ± 3.80	7.94 ± 0.61	44.72 ± 1.06	20.35 ± 2.85	37.29 ± 3.92	19.85 ± 0.99
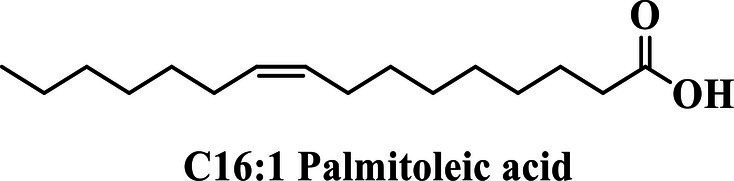	4.48 ± 0.26	2.14 ± 0.30	5.92 ± 1.77	2.63 ± 0.49	5.01 ± 0.94	2.65 ± 0.18
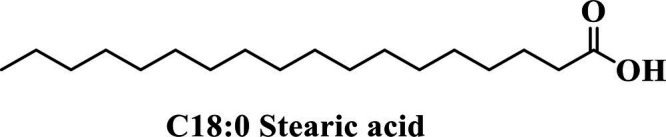	0.00 ± 0.00*	0.00 ± 0.00*	6.64 ± 1.81	2.99 ± 0.74	2.35 ± 2.10	1.33 ± 1.24
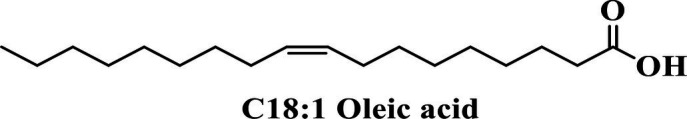	137.01 ± 23.47	64.39 ± 0.22	111.90 ± 19.21	50.13 ± 2.79	96.52 ± 13.95	51.21 ± 1.70
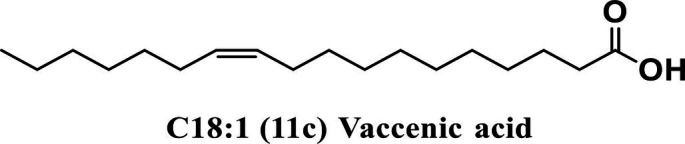	9.16 ± 1.15	4.35 ± 0.49	10.16 ± 1.85	4.56 ± 0.46	7.61 ± 0.75	4.05 ± 0.19
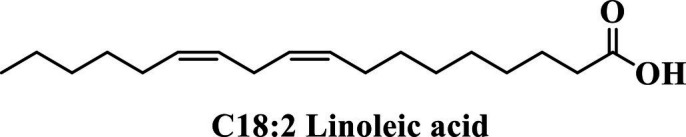	n.d	n.d	n.d	n.d	n.d	n.d
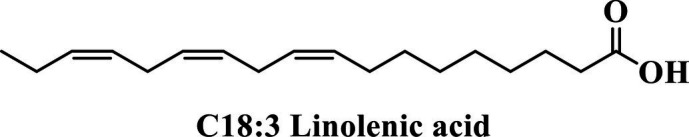	32.89 ± 4.75	15.52 ± 0.73	31.95 ± 4.80	14.36 ± 0.87	28.40 ± 2.69	15.13 ± 0.64
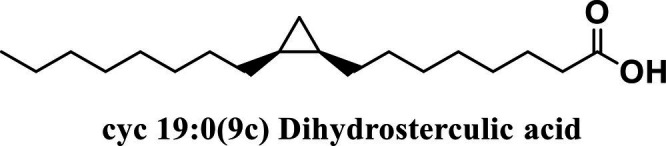 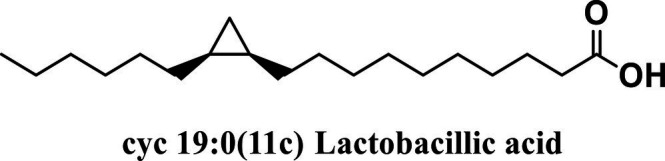	n.d	n.d	n.d	n.d	n.d	n.d
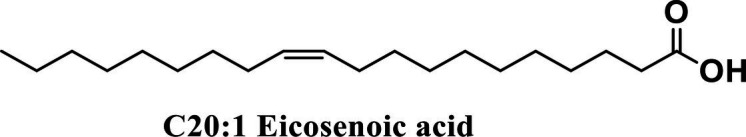	9.93 ± 1.78	4.66 ± 0.16	9.39 ± 0.59	4.25 ± 0.29	9.57 ± 1.46	5.08 ± 0.47
Total FA (μg FAME/mg CDW)	212.81 ± 24.43^a^		222.22 ± 20.13^a^		188.09 ± 15.05^a^	
UFA (μg FAME/mg CDW)	193.46 ± 24.04		169.32 ± 19.97		147.11 ± 14.33	
SFA (μg FAME/mg CDW)	19.35 ± 4.33		52.90 ± 2.51		40.98 ± 4.60	
UFA:SFA	10.00^a^		3.20^b^		3.59^b^	

* Detectable but below the limit of quantification.

Relative abundance = (Fatty acid/total fatty acids detected)*100. Data are mean ± SD of three biological replicates for each of the three sparging conditions. One-way ANOVA (Tukey’s multiple comparison test) to determine statistical significance. Equal letters indicate no statistical difference between the samples (value of *p* > 0.05). CDW, cell dry weight; FA, fatty acid; FAME, fatty acid methyl ester; UFA, unsaturated fatty acid; SFA, saturated fatty acid; n.d, not detected.

## Discussion

Industrial production of probiotics needs to tolerate adverse environmental conditions to attain a stable product (Fenster et al., 2019). Oxygen is known to inhibit the growth of *L. reuteri* (Rao et al., 2021) and is typically curtailed in cultivation processes. Here, we demonstrate that providing oxygen at levels low (<500 mL/min) enough to not inhibit growth resulted in a higher FD stress tolerance of *L. reuteri* (Figure 4). This may be explained by a higher level of unsaturated over saturated fatty acids (Table 2), possibly making the phospholipid membrane fluidity higher, similarly as observed previously for other microorganisms (Hua et al., 2009; Li et al., 2009; Fonseca et al., 2019). Furthermore, air sparging was found to give a slight improvement in the bile tolerance, while the acid tolerance was lower compared to anaerobic condition. The overall positive effects of low levels of oxygen on the probiotic product implies that some degree of aeration should be applied during cultivation of *L. reuteri*; however, the generality of this strategy needs to be further investigated also for other *L. reuteri* strains than DSM 17938, since oxygen tolerance is a strain-specific property (Guilbaud et al., 2012).

Air sparging at 500 mL/min did not impact the growth significantly, hence could be concluded that it could be supplied without consequences for biomass production (Table 1). This might suggest that the level of aeration utilized during the experiments were not high enough to bring about a change in terms of growth. It should be noted, however, that the presence of air significantly increased the amount of acetate produced, at the cost of reduced ethanol and lactate. In the presence of oxygen, NADH is oxidized to NAD^+^ by NADH oxidase (Higuchi et al., 2000). This aids in reducing the cellular redox imbalance by allowing part of the formed acetyl phosphate to be converted into acetate with a co-generation of ATP, and reduces the flux toward the NADH oxidation step in which ethanol is formed (Supplementary Figure S3). For the non-sparged and N_2_ sparged cultivations, high amounts of ethanol were seen which suggests that NADH is more abundant (inactive/low activity of NADH oxidase), and ethanol is formed *via* the phosphoketolase pathway.

Another pronounced metabolic difference was the lack of citrate assimilation under the anaerobic condition. *Limosilactobacillus reuteri* 55730 (mother strain of DSM 17938) has a partial TCA cycle, which enables citrate to be utilized as an external electron acceptor (Saulnier et al., 2011). A study by Kaneuchi et al. (1988) showed that 23 out of 39 *L. reuteri* strains converted citrate to succinate. However, we did not observe succinate formation by DSM 17938 in the present study. It is likely that citrate is initially converted to oxaloacetate and acetate by the enzyme citrate lyase (Supplementary Figure S3) and then further to pyruvate by oxaloacetate decarboxylase, and to lactate by lactate dehydrogenase (Hugenholtz, 1993).

A small but detectible difference was observed in the morphology of cells between the 3 conditions (Figure 2). Differences in cell size during batch cultivations (different time points) has been observed before in *Escherichia coli* (Kaganovitch et al., 2018). In our previous study (Rao et al., 2021), there was evidence that *L. reuteri* displays pleomorphic behavior when subjected to different environmental conditions and this might be due to the improper cell division cycle or differences in membrane composition (Weart et al., 2007; van Bokhorst-van de Veen et al., 2011).

There is still a question on how UFA are formed in *L. reuteri* DSM 17938 since there was no indication of *L. reuteri* having a fatty acid desaturase, based on the current gene annotations and the protein domain analysis (Supplemental material S2). However, in several bacterial species, the enzyme fabA (β-Hydroxydecanoyl-ACP dehydratase—has two functions, dehydratase and isomerase) isomerises trans-2-decenoyl-ACP to cis-3-decenoyl-ACP, thereby by-passing fabI (enoyl reductase), and allow biosynthesis of unsaturated fatty acids (Heath and Rock, 1996; Keweloh and Heipieper, 1996). The protein domain analysis identified two *L. reuteri* SD2112 genes containing fabA/fabZ domains (Supplementary material S2), and thus it is tempting to speculate that the observed differences are related to lower availability of NADH in presence of oxygen. A limitation of NADH may result in less efficient reduction of enoyl-ACP to fatty acyl-ACP, which is carried forward by the enzyme fabI in the elongation cycle of the fatty acid biosynthesis (Supplementary materials S1, S2; Supplementary Figure S2). Another plausible explanation is different contribution of unsaturated fatty acids from the extracellular surrounding. MRS medium used herein contains components such as Tween 80®, which is a surfactant that is composed of 60% oleic acid, as well as yeast extract and beef extract. These components add to the complexity to deduce if there is *de novo* fatty acid synthesis or if these components directly get access to the phospholipid bilayer. A study by Reitermayer et al. (2018) exhibited that *L. plantarum* TMW 1.708 grown on MRS media in 96-well plate concluded that Tween 80® contributes by conserving energy that is needed for *de novo* fatty acid synthesis to improve growth. Although no clear evidence has been shown about the incorporation of Tween 80® into cell membrane. A clean medium is required to study the *de novo* fatty acid synthesis.

Nitrogen sparged cultivation displayed better tolerance to low pH when compared to air sparged cultivation. This could be due to the increased function/activity of the glutamate decarboxylase (GAD) during anaerobic conditions that is associated with the acidification of both the medium and the intracellular environment that takes place during the process of fermentation (Sewell et al., 2015). GAD induction could initiate acid tolerance response (Feehily and Karatzas, 2013; Teixeira et al., 2014; Papadimitriou et al., 2016). The impact of oxygen on the resistance of bile was studied. It displayed higher tolerance to bile when cultivated in air compared to other conditions. A study by Wright et al. (2016), displayed that oxygen availability does influence resistance in *L. monocytogenes,* but the mechanism by which it does is still not clear and needs further investigation. Oxygen stress induces higher activity of 5′ NT which has been displayed previously (Pang et al., 2022) which could suggest higher bioactivity (production of adenosine).

Here, it can be concluded that low levels of oxygen did not influence the growth rate and biomass yield, but resulted in modulation of redox metabolism, increased the UFA:SFA ratio for cells grown in MRS medium, which may have contributed to the observed improvement in FD survivability in DSM 17938. It displayed improved bile tolerance, higher 5′ nucleotidase activity but reduced tolerance to low pH.

## Data availability statement

The original contributions presented in the study are included in the article/Supplementary material, further inquiries can be directed to the corresponding authors.

## Author contributions

NR designed the experiments, performed majority of experiments, analyzed the data, and drafted the manuscript. LE, JT, CT, DB, SP, EN, and SH helped and coordinated in the entire study. MC provided the resources, supervised, and did major review of the manuscript. All authors contributed to the article and approved the submitted version.

## Funding

The study was partly financed by BioGaia AB.

## Conflict of interest

LE, JT, and SH were employed by BioGaia AB.

The remaining authors declare that the research was conducted in the absence of any commercial or financial relationships that could be construed as a potential conflict of interest.

## Publisher’s note

All claims expressed in this article are solely those of the authors and do not necessarily represent those of their affiliated organizations, or those of the publisher, the editors and the reviewers. Any product that may be evaluated in this article, or claim that may be made by its manufacturer, is not guaranteed or endorsed by the publisher.

## Footnotes

^1^ https://www.ncbi.nlm.nih.gov/
