# Supplementary material for: Non-inhibitory levels of oxygen during cultivation increase freeze-drying stress tolerance in Limosilactobacillus reuteri DSM 17938
Source: Front Microbiol. 2023 Apr 14;14:1152389. doi: 10.3389/fmicb.2023.1152389 (PMC10140318; doi:10.3389/fmicb.2023.1152389)
Supplement: Supplementary file 1 [file Data_Sheet_1.docx]

**Supplementary material S1:**

**Detailed description of the flow cytometry gating strategy**

The purpose of the study was to establish a flow cytometry workflow using an automated sampling unit coupled with automated gating that would be less labour intensive, less time consuming, and less biased in data processing compared to manual flow cytometry workflows. The gating strategy was developed using on *Limosilactobacillus reuteri* data from our previous study (Rao *et al.*, 2021); in total, data from five different cultivation conditions were used. Three gating strategies were compared: a manual gating strategy where gates were user-defined using a graphical interface and gates were set using the mouse; a fixed gating strategy where the data was processed by a script and gates were user-defined based on x- and y-coordinates and linear equations; an automated gating strategy where the data was processed by a script and gates were set based on a *k*-means cluster analysis of the data. The raw data from the flow cytometer were exported as FCS3.1 files. The Matlab scripts used to process and analyze the flow cytometry data is available on the following GitHub page: <https://github.com/MicrobialEngineeringGroupTMB/>.

**Manual gating strategy**

The FCS files were processed using the FlowJo v10.7 software (FlowJo, LLC, USA). Viability was studied using cells stained with a mix of two dyes: SYBR green and propidium iodide (PI). SYBR green binds to DNA of all the cells and PI is extensively used to evaluate cell membrane integrity. An initial gate was set based on the scattering profiles (FSC and SSC) to remove the electronic noises. The remaining events were analysed using a log scale density plot of FL1-H vs FL3-H, to visualize the fluorescence intensity of the dyes. Gating was performed based on previous work by Nescerecka et al., 2016 where the cells were gated into two sub-populations, intact and damaged cells. Two distinct subpopulations within the damaged cells were also observed, and were thus labelled as Damaged cells A and Damaged cells B. Any events falling outside these three populations were gated as cell debris, due to their low FL1-H and FL3-H values (Figure S1-1).


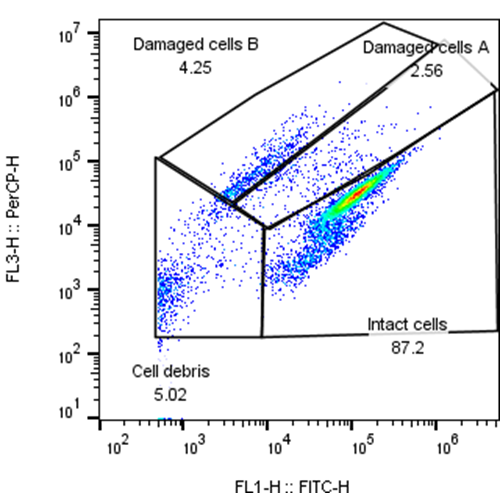


**Figure S1-1:** Example of the manual gating strategy for identification of subpopulations in the *L. reuteri* samples, using the FlowJo v10.7 software.

**Fixed gating strategy**

The second gating strategy investigated in the study was fixed gating and was performed using MATLAB R2019a. FCS files were loaded in MATLAB using the *fca_readfcs* script (Balkay, 2023). A pipeline for clean-up and gating was established (Figure S1-2). The clean-up consisted of setting lower threshold values for on FL1-H and FL3-H channels and was performed to remove noise from the data (black vertical and horizonal lines in Figure S1-2). Two linear equations were then used to separate subpopulations according to the same definitions used in the manual gating strategy (Figure S1-1): one equation parted damaged B and damaged A subpopulations and one equation parted the intact and damaged A subpopulations. Microsoft Excel (Microsoft, Redmond, WA) was used to generate equations from the FL1-H and FL3-H dot plot of a representative sample. The boundaries of the resulting gates are represented with blue stars in Figure S1-2. The fixed gates were then applied to all samples from each run.


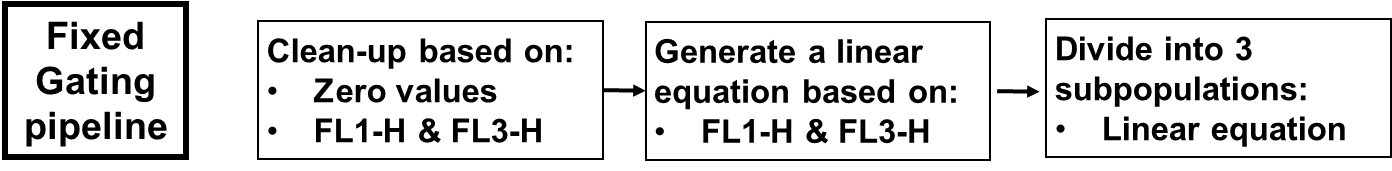


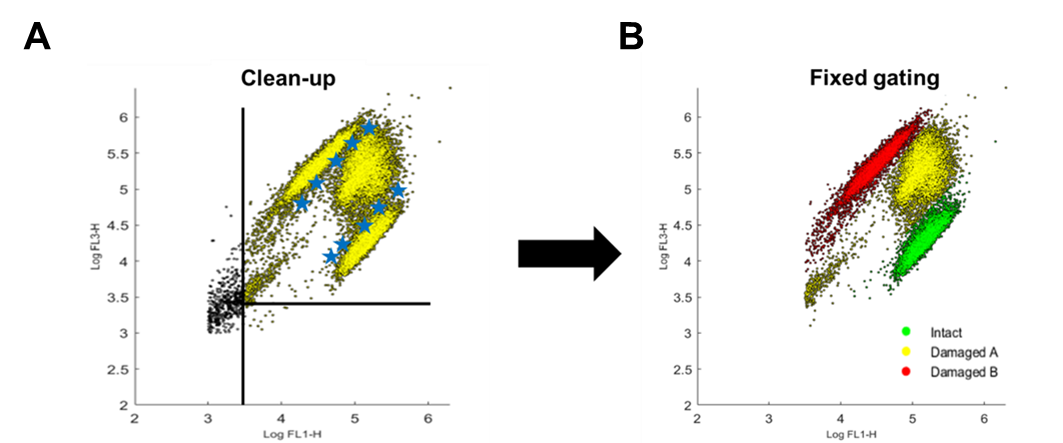


**Figure S1-2:** Flow chart for fixed gating strategy. (A) The clean-up of data was based on absolute limits set on FL1-H and FL3-H as well as removal of events with any variable with zero value. (B) The events were divided into live, damaged A cells, damaged B cells by acquiring a suitable linear equation using a dot plot (FL1-H vs FL3-H). The blue stars signify points chosen manually to generate an equation.

***k*-means clustering algorithm**

An automated data processing work-flow was created based on *k*-means clustering. This is an unsupervised clustering method in which the number of clusters (*k*) is defined by the user. In the initial step of the iterative clustering algorithm, *k* random points are inserted as initial centroids into the data from a random seed. The *k* clusters are created after each flow cytometry event in the dataset is allocated to the closest centroid. The centroids are then recalculated depending on the actual cluster centre point for each iteration. In the second phase, each event will be reassigned if doing so would minimise the sum of distance to the centroid. Following each reassignment, new centroids are calculated. After all events have been evaluated, a local minimum of the smallest sum of distance is found; other local minimums may be discovered depending on the location of the initial seeds for the centroids. The process is then repeated with new seeds, and the final clustering results are determined by the best sum of distances. A limitation of the implementation of the *k*-means algorithm used here was that the number of clusters must be pre-defined. In this study, it was found that the best clustering results were obtained by setting *k* =3 to try to cluster intact cells, damaged cells A and damaged cells B ( Figure S1-3). The final cluster, i.e. the cell debris, was then identified by further gating of the damaged cells A subpopulation: the FL1-H channel data plotted as a histogram and the a gate was set by calculating the global minimum and designating all events below that value as cell debris (Figure S1-3).


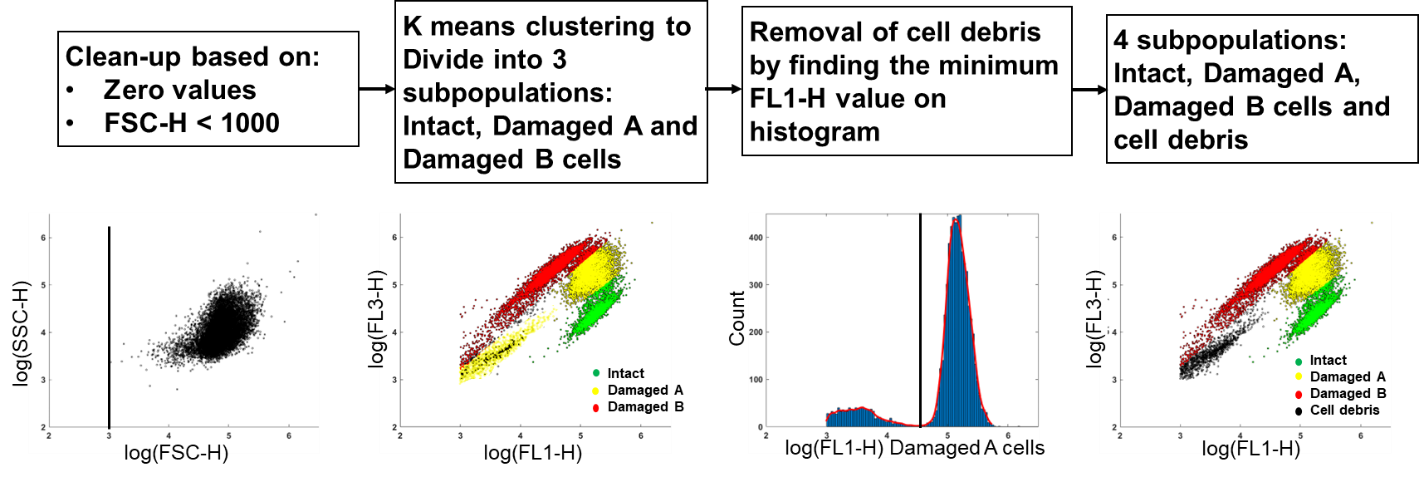


**Figure S1-3:** Flow chart for *k*-means clustering gating strategy. (i) Clean-up of the noise was based on removal of any event with zero variable as well as a set limit on FSC-H for 1000, (ii) Three clusters were assigned using the MATLAB script for each sample obtaining three distinct populations (Intact, Damaged A and Damaged B cells), (iii) To remove the cell debris, FL1-H histogram of damaged A cell population was used. The MATLAB function *min* was used to find the global minimum value and events below that limit was labelled as cell debris. (iv) This generated 4 subpopulations.

**Evaluation of the different gating strategies**

To benchmark the different gating strategies, each FCS file were in turn subjected to all three gating strategies. The outcome of the different strategies were evaluated based on evaluating the identified total cell counts and cell counts for each subpopulation by comparing the manual gating strategy to either the fixed or the *k-*means gating strategy (Table S1). It was found that the *k*-means gating strategy performed better than the fixed gating when it came to the damaged cells A and B gates.

Manual gating is time-consuming, and impractical for large amounts of samples as the same gates cannot be applied for all the samples and needs to be dynamically altered according to the profile of each sample on the desired channels. Out of the two script-based gating strategies analysed in this study, the *k*-means clustering strategy was found to be less biased and adapts to the sample changes. The major downside of the type of fixed gating strategy used in this study was that the equations of the gates needed to be computed manually by picking the points in the FL1-H vs FL3-H plot. The same equation-based gates were applied to all samples, which also goes against the idea discussed in related to the manual gating that each sample needs to be gated individually. In this way, this type of fixed gating is just as prone as manual gating to variation between users. Some limitations of the *k*-means algorithm when analysing flow cytometry data is that the number of clusters must be pre-defined, the populations are limited to a spherical or elliptical shape of clusters as well as the sensitivity to the seed points of initial centroid locations.

**Table S1:** Correlation between manual gating (FlowJo software) and fixed gating, K-means clustering for total cell count and individual subpopulations. Correlation coefficient (R^2^) was computed by using the function *RSQ* on Microsoft excel.

|  | **Correlation coefficient (R^2^)** | | | |
| --- | --- | --- | --- | --- |
|  | **Total cell count** | **Intact cell count** | **Damaged A cell count** | **Damaged B cell count** |
| **Manual gating method vs Fixed gating** | 0.999 | 0.999 | 0.892 | 0.956 |
| **Manual gating method vs K-means clustering** | 0.999 | 0.999 | 0.942 | 0.950 |

**Supplementary figures**

**
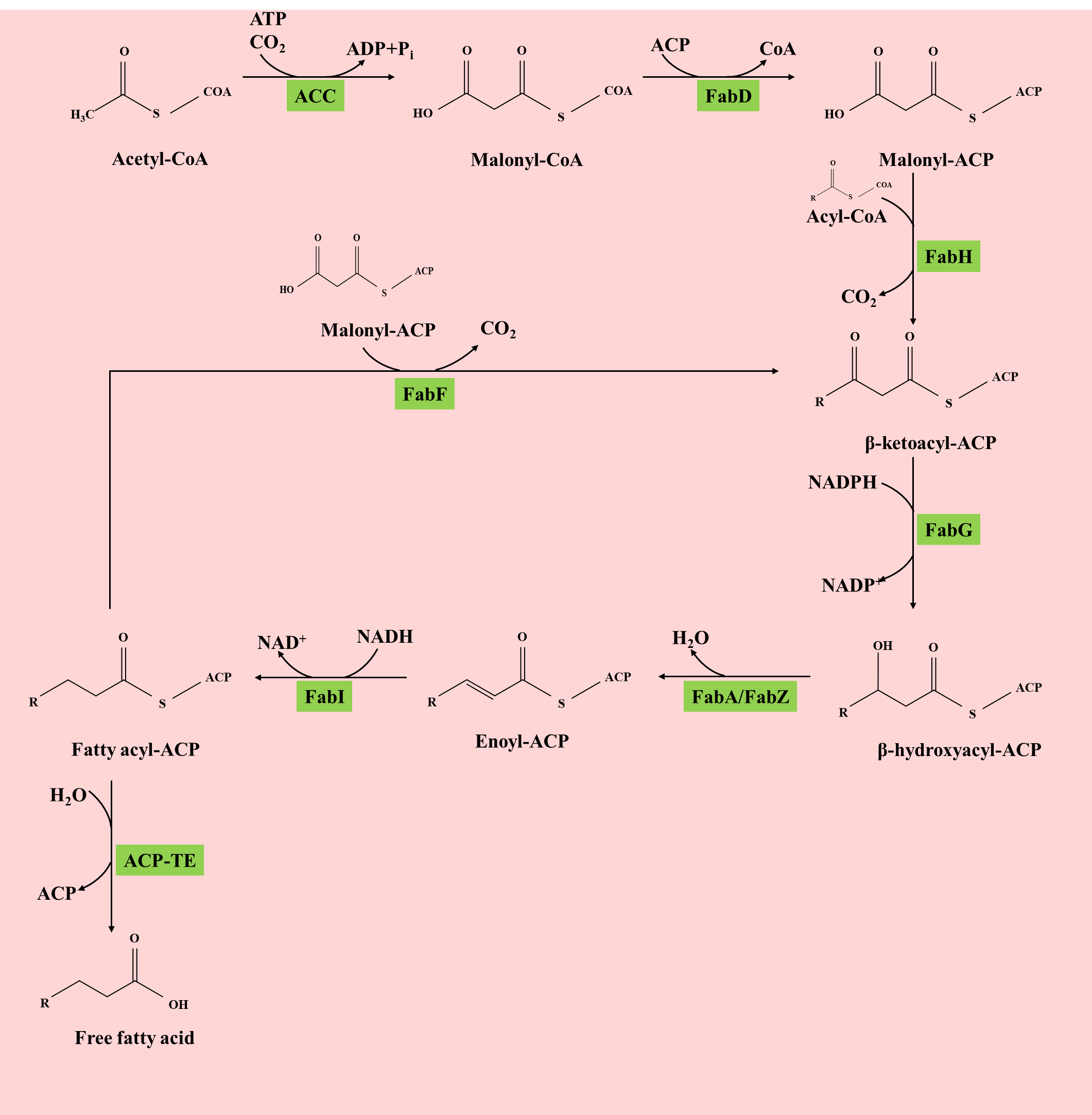
**

**Figure S2:** Formation of free fatty acid in bacteria. The initiation phase of type II fatty acid synthesis (FASII). Acetyl-CoA carboxylase (ACC) is composed of four subunits and provides malonyl-CoA elongation units to the elongation cycle. The malonyl groups are transferred to the acyl carrier protein (ACP) by malonyl- CoA–ACP transacylase (FabD). β-ketoacyl-ACP synthase III (FabH) is the initiating condensing enzyme that is primed by an acyl-CoA to condense with malonyl-ACP to start a new fatty acid. The elongation of fatty acid chains is an iterative process that involves 2-carbon building blocks. The β-ketoacyl-ACP intermediate is reduced by the FabG β-ketoreductase, and the β-hydroxyacyl-ACP is dehydrated to enoyl-ACP by FabA or FabZ. The cycle is pulled to completion by the enoyl-ACP reductase (FabI). New rounds of elongation are initiated by the elongation condensing enzymes (FabF). Once a desired chain length is achieved, acyl-ACP thioesterase (ACP-TE) catalyses the hydrolysis of thioester bonds and directly determines fatty acid chain length.


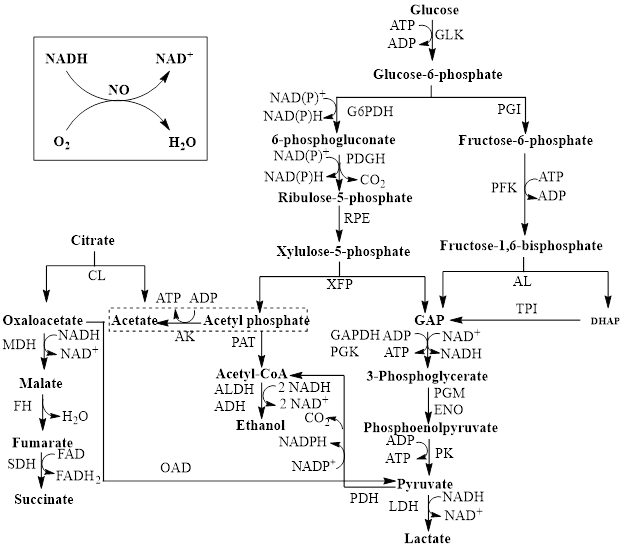


**Figure S3:** Pathway of glucose metabolism in *L. reuteri* DSM 17938 (phosphoketolase pathway (PKP) and Embden–Meyerhof–Parnas (EMP) pathway). GLK, glucokinase; G6PDH, glucose-6-phosphate dehydrogenase; PGI, Phosphoglucose isomerase; PFK, Phosphofructose kinase; PDGH, 6-phosphogluconate dehydrogenase; RPE, ribulose phosphate 4-epimerase; XFP, Xylulose 5-phosphate phosphoketolase; AL, Aldolase; TPI, Triose phosphate isomerase; GAPDH, Glyceraldehyde phosphate dehydrogenase; PGK, phosphoglycerate kinase; PGM, phosphoglycerate mutase; ENO, enolase; PK, pyruvate kinase; LDH, lactate dehydrogenase; PAT, phosphate acetyltransferase; ALDH, acetaldehyde dehydrogenase; ADH, alcohol dehydrogenase; AK, acetate kinase; CL, citrate lyase; MDH, malate dehydrogenase; FH, fumarate hydratase; SDH, succinate dehydrogenase; OAD, oxaloacetate decarboxylase; PDH, pyruvate dehydrogenase; NO, NADH oxidase.

**Table S2: Viable cell count before and after freeze drying (FD). CFU, colony forming units.**

| **Conditions** | **Viable cell counts before FD** **(CFU/ml)** | **Viable cell counts after FD (CFU/ml)** |
| --- | --- | --- |
| **Air sparged** | 7.86x10^9^ ± 7.40x10^8^ | 4.57x10^9^ ± 5.04x10^8^ |
| **Non-sparged** | 1.70x10^10^ ± 3.10x10^9^ | 1.01x10^10^ ± 1.15x10^9^ |
| **N_2_ sparged** | 9.90x10^9^ ± 8.25x10^8^ | 1.17x10^9^ ± 5.02x10^8^ |

**References**

Balkay, L. (2023). fca_readfcs, MATLAB Central File Exchange, <https://www.mathworks.com/matlabcentral/fileexchange/9608-fca_readfcs>, Retrieved January 20, 2023.

Nescerecka, A., Hammes, F. and Juhna, T. (2016) ‘A pipeline for developing and testing staining protocols for flow cytometry, demonstrated with SYBR Green I and propidium iodide viability staining’, *Journal of Microbiological Methods*. Elsevier B.V., 131, pp. 172–180. doi: 10.1016/j.mimet.2016.10.022.

Rao, N. S. *et al.* (2021) ‘Flow cytometric analysis reveals culture condition dependent variations in phenotypic heterogeneity of Limosilactobacillus reuteri’, *Scientific Reports*. Nature Publishing Group UK, 11(1), p. 23567. doi: 10.1038/s41598-021-02919-3.
